# Design of an Artificial Intelligence of Things-Based Sesame Oil Evaluator for Quality Assessment Using Gas Sensors and Deep Learning Mechanisms

**DOI:** 10.3390/foods12214024

**Published:** 2023-11-03

**Authors:** Hao-Hsiang Ku, Ching-Fu Lung, Ching-Ho Chi

**Affiliations:** 1 Institute of Food Safety and Risk Management, National Taiwan Ocean University, Keelung City 202301, Taiwan; 2 Department of Food Science, National Taiwan Ocean University, Keelung City 202301, Taiwan; 0093a005@mail.ntou.edu.tw; 3 Institute of Clinical Pharmacy and Pharmaceutical Sciences, National Cheng Kung University, Tainan City 701401, Taiwan; s68091029@gs.ncku.edu.tw

**Keywords:** sesame oil, artificial intelligence of things, artificial neural network, convolutional neural network, long short-term memory, deep learning

## Abstract

Traditional oil quality measurement is mostly based on chemical indicators such as acid value, peroxide value, and *p*-anisidine value. This process requires specialized knowledge and involves complex steps. Hence, this study designs and proposes a Sesame Oil Quality Assessment Service Platform, which is composed of an Intelligent Sesame Oil Evaluator (ISO Evaluator) and a Cloud Service Platform. Users can quickly assess the quality of sesame oil using this platform. The ISO Evaluator employs Artificial Intelligence of Things (AIoT) sensors to detect changes in volatile gases and the color of the oil during storage. It utilizes deep learning mechanisms, including Artificial Neural Network (ANN), Convolutional Neural Network (CNN), and Long Short-Term Memory (LSTM) to determine and evaluate the quality of the sesame oil. Evaluation results demonstrate that the linear discriminant analysis (LDA) value is 95.13. The MQ2, MQ3, MQ4, MQ7, and MQ8 sensors have a positive correlation. The CNN combined with an ANN model achieves a Mean Absolute Percentage Error (MAPE) of 8.1820% for predicting oil quality, while the LSTM model predicts future variations in oil quality indicators with a MAPE of 0.44%. Finally, the designed Sesame Oil Quality Assessment Service Platform effectively addresses issues related to digitization, quality measurement, supply quality observation, and scalability.

## 1. Introduction

In recent years, a significant rise in food safety incidents, including melamine, plasticizers, contaminated starch, toxic soy sauce, recycled cooking oil, and tainted eggs, has brought about substantial threats and risks to human life and health [1,2,3]. As a result, there has been an increased emphasis on food safety. Oil products, being an integral component of daily consumption, can have widespread implications if their quality is compromised. Therefore, it is crucial to exercise vigilance and promptly detect any changes in oil quality before consumption. Among various oil products, flavored oils play a vital role in enhancing the taste of culinary preparations. Sesame oil, specifically used in Asian cuisine, stands out due to its unique nutritional composition and distinct flavor profile, setting it apart from other oils. Furthermore, extensive research has shown that sesame oil contains a higher concentration of antioxidants compared to most other oil products. The sesame oil has been stored for an extended period, and oxidation indicators, such as peroxide value and *p*-anisidine value, are noticeably lower than those of other oil products. This suggests that sesame oil is more effective in antioxidation [4]. Therefore, the topic of developing a simple method for the public to assess the quality of sesame oil is a worthy research subject.

Lipid oxidation poses a significant food safety concern, particularly in the context of edible oils. This process not only leads to the deterioration of the sensory properties of oil products but also results in the formation of compounds like hydroperoxides and aldehydes, which can have adverse health effects on consumers [5,6]. To gauge the freshness and safety of edible oils, peroxide values are often used as a critical indicator. Fresh oils typically exhibit peroxide values below 10 meq/kg, while values exceeding 100 meq/kg have been associated with food poisoning incidents [7]. The need for accurate and efficient methods to monitor edible oil quality has prompted the adoption of advanced technology, where researchers in the food industry are exploring the integration of information technologies into traditional measurement methods [8]. Therefore, to assess the quality of oil products, related studies have been roughly classified into three distinct methodologies.

(1) Chemical analyses of oils: In the assessment of the oxidative stability of sesame oil, extracted using different methods or obtained through roasting processes at various temperatures; commonly used chemical indicators include acid value (AV), peroxide value (POV), and *p*-anisidine value (AnV) [9,10,11,12]. These indicators are assessed using a variety of techniques, including paper-based analytical devices (PADs) employing an iodometric method [13], room-temperature phosphorescence (RTP)-based sensors [14], and spectroscopies like near-infrared (NIR), mid-infrared absorption, and Raman scattering combined with partial least squares regression (PLSR) for quick and sensitive POV estimation [15,16]. Additionally, techniques such as surface-enhanced Raman spectroscopy (SERS) [17], spectrophotometric iodide-dependent methods [18], and chromatography methods (GC-FID and HPLC-ELSD) are employed to measure various aspects of edible oil quality and authenticity [19].
(2) Data Analysis: Researchers employ various methods to assess the quality and authenticity of edible oils. Liu et al., use Terahertz spectra to evaluate peanut oil’s peroxide value (POV) and employ genetic algorithms (GA) and principal component analysis (PCA) to process THz data, ultimately achieving better correlation between absorbance spectra and POV [20]. Surya et al., implement Long Short-Term Memory (LSTM) with the seagull optimization algorithm (SOA) for improved classification and authenticity assessment, outperforming existing techniques [21]. Dou et al. utilize headspace gas chromatography–ion mobility spectrometry (HS-GC-IMS) to detect sesame oil adulteration effectively, distinguishing authentic and counterfeit products using chemometric methods [22]. Meng et al., apply Fourier-transform infrared (FTIR) and visible–near-infrared (Vis-NIR) spectroscopy in detecting adulterated olive oil, achieving high accuracy and a user-friendly technique [23]. Liu et al., develop a visual array sensor for sesame oil adulteration detection, reaching a 100% classification accuracy [24]. Chen et al., create a model for detecting sesame oil adulteration using fatty acids, phytosterols, and tocopherols and employ chemometric techniques for effective screening and verification [25]. These methods involve the utilization of headspace gas chromatography–ion mobility spectrometry (HS-GC-IMS) and various spectroscopic techniques, along with analytical algorithms, such as Terahertz spectra and Long Short-Term Memory (LSTM), combined with chemometrics for identifying adulteration and assessing oil quality.
(3) Internet of Things Technology: Researchers have harnessed electronic nose (E-nose) technology to enhance the detection of adulteration and assess the quality of edible oils. Aghili et al., effectively employed E-nose in conjunction with chemometric methods to detect low-level fraud in vegetable oils, presenting a promising approach for improving efficiency and ensuring safety [26,27,28]. Zarezadeh et al., fused E-nose with ultrasound methods, achieving a significant increase in classification accuracy. Their artificial neural networks (ANN) method reached an impressive 95.51% accuracy in identifying fraudulent olive oil samples [29]. Han et al., applied E-nose alongside gas chromatography–ion mobility spectrometry (GC-IMS) to differentiate oils and detect adulteration in safflower seed oil (SSO), making a valuable contribution to SSO quality assessment [30]. Xing et al., discussed the potential of E-nose as a low-cost, portable, and sensitive technology for food quality assessment, addressing challenges and prospects in odor-based food safety and quality monitoring [31]. Karami et al., used E-nose in combination with ANN and PCA to predict the shelf life and oxidation degree of oils, highlighting the effectiveness of ANN and E-nose in pattern recognition and quality assessment [32]. Anwar et al., explored the use of electronic nose technology and machine learning algorithms for rapid and cost-effective food quality assessment across various food categories, providing case studies demonstrating its potential to enhance food industry evaluation [33]. Teixeira et al., employed E-nose alongside PCA and linear discriminant analysis (LDA) to classify olive oils based on fruity intensity, offering a non-destructive tool for quality assessment, particularly for extra virgin olive oils [34]. These IoT-based approaches are delivering rapid, cost-effective, and efficient solutions for enhancing food quality assessment, monitoring, and safety in the food industry.

Taking into account the above-mentioned approaches when designing a sesame oil quality evaluator, it is important to consider the following issues: (1) digitization, (2) quality assessment, (3) quality monitoring, and (4) scalability. Each of these aspects is described below:

(1) Digitization: The device should incorporate digital technologies to enhance the efficiency and accuracy of testing. This could involve using sensors, data analysis algorithms, and connectivity capabilities to gather and process relevant information.
(2) Quality assessment: The evaluator should be able to assess the quality of sesame oil based on established standards and parameters. This may involve analyzing various factors such as acid value, peroxide value, presence of contaminants, and sensory attributes like aroma and flavor.
(3) Quality monitoring: The evaluator should facilitate monitoring the quality of sesame oil throughout the supply chain. This could involve implementing quality control measures at different stages, including production, processing, storage, and distribution, to ensure consistent and safe products.
(4) Scalability: The evaluator should be designed to accommodate potential future needs and advancements in technology. It should be flexible and adaptable, capable of integrating new features and functionalities as the field of smart testing evolves.

By addressing these issues, the development of a sesame oil quality evaluator can provide a convenient and reliable method for the public to assess the quality of sesame oil and promote consumer confidence in its safety and authenticity.

The remainder of this study is organized as follows. Section 2 describes the system framework and components of the Sesame Oil Quality Assessment Service Platform. Section 3 describes the implementation and evaluations. Finally, Section 4 gives the concluding remarks.

## 2. The System Framework of Sesame Oil Quality Assessment Service Platform

**Figure 1:**
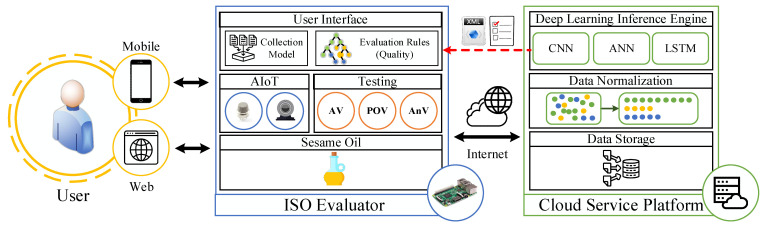
The framework of the Sesame Oil Quality Assessment Service Platform.

The Sesame Oil Quality Assessment Service Platform is composed an Intelligent Sesame Oil Evaluator (ISO Evaluator) and a Cloud Service Platform. Users can quickly assess the quality of the sesame oil using this platform. The core mechanism of the ISO Evaluator is based on the integration of AIoT and deep learning technologies. The ISO Evaluator utilizes AIoT devices to collect volatile compounds emitted by sesame oil and captures images of the color status of sesame oil using a camera. The collected data are securely stored on a data storage to ensure data accuracy and enable traceability. Subsequently, a deep learning module analyzes the data to obtain the quality assessment of the sesame oil. The overall architecture of the system consists of two main components: (1) the construction of the Intelligent Sesame Oil Evaluator and (2) the establishment of a Cloud Service Platform. The detail framework is shown in Figure 1.

### 2.1. Intelligent Sesame Oil Evaluator

**Figure 2:**
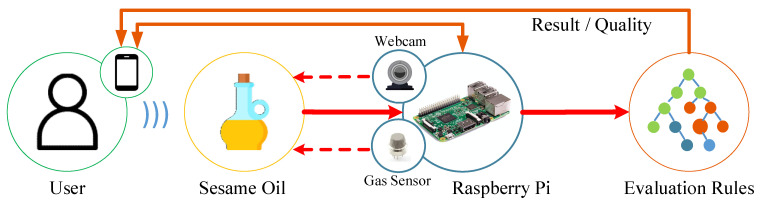
The working flow of the ISO Evaluator.

The Intelligent Sesame Oil Evaluator (ISO Evaluator) leverages AIoT devices, gas sensors, and a web camera to capture and monitor the relevant characteristics of sesame oil. The collected data, including the volatile compounds and color images, are processed in real-time to provide an immediate assessment of the oil’s quality. The working flow is shown in Figure 2.

#### 2.1.1. AIoT Control Module

The AIoT Control Module is built on a Raspberry Pi. It is responsible for automating the control and recording of multiple gas sensors, which is equipped with various gas sensors, including MQ2, MQ3, MQ4, MQ5, MQ6, MQ7, MQ8, MQ9, MQ135, and MQ137, to detect volatile compounds present in sesame oil. These compounds include pyrazines, pyrroles, furans, carbonyls, and sulfur-containing compounds. The detected gases include ethanol, methane, carbon monoxide, hydrogen, hydrogen sulfide, ammonia, carbon dioxide, and so on. It also identifies volatile compounds such as aldehydes, alcohols, ketones, and others. In addition, it also integrates a webcam for capturing sesame oil images to record quality and for visual recognition to confirm the quality.

#### 2.1.2. Oil Quality Indicator Collection Module

The oil quality indicator collection module is used to collect various indicators that determine the quality of the oil. It is designed to gather data related to the acid value, peroxide value, and *p*-anisidine value of the oil. These indicators provide important information about the freshness, stability, and overall quality of the oil. The module aims to systematically collect and store this data to establish a comprehensive oil quality database for analysis and comparison purposes.

(a) The acid value (AV) is a measure of the amount of potassium hydroxide (KOH) in milligrams (mg) required to neutralize the free fatty acids present in 1 gram of oil or fat. This indicator is used to assess the content of free fatty acids in the oil or fat, which represents the extent of acid hydrolysis of the oil or fat. It also reflects the quality of the oil or fat, as fresher oils or fats tend to have a lower acid value. According to the specifications and standards set by the Codex and the Taiwan Quality Food Association (TQF) for edible oil and fat inspection, the acid value requirement for pressed sesame oil should be below 4 mg/g [35,36].
(b) The peroxide value (POV) is the milliequivalent of active oxygen per kilogram of oil or fat. Hydroperoxides are the primary products of oil or fat oxidation, and during the initial stages of oxidation, the POV increases as the oxidation progresses. However, when the oil or fat is highly oxidized, the decomposition rate of hydroperoxides exceeds their formation rate, leading to a decrease in POV. Therefore, POV is suitable for measuring the initial stage of oxidation in oil or fat. According to the specifications and standards set by the Codex and the Taiwan Quality Food Association (TQF) for edible oil and fat inspection, the peroxide value requirement for pressed sesame oil should be below 15 milliequivalents per kilogram (meq/kg) [35,36].
(c) The *p*-anisidine value (AnV) is an indicator used to measure the secondary oxidation products in oils and fats, specifically as an indicator of the late stages of oxidation. During the initial stages of oil or fat oxidation, hydroperoxides are formed, which then undergo decomposition and polymerization to form aldehydes, ketones, acids, and other compounds. Aldehyde compounds react with *p*-anisidine reagent, and the absorbance is measured at a wavelength of 350 nm [37]. The higher the absorbance value, the greater the presence of aldehyde compounds, and the higher the degree of oxidation. Therefore, the *p*-anisidine value serves as an indicator of the extent of oxidation in oils and fats.

Once the oil quality indicator collection module has collected the aforementioned three indicators, it will integrate the data from the gas sensors and the color analysis of sesame oil for the same batch. The module will then transmit these data to a cloud server, where they can be used to establish or adjust the accuracy of evaluation criteria. By analyzing the combined data from the gas sensors and color analysis, the cloud server can develop or refine assessment rules that accurately reflect the quality of the oil. This integration of data and cloud-based analysis helps in enhancing the accuracy of evaluating the oil quality.

#### 2.1.3. Evaluation Module

The evaluation module is responsible for assessing the quality of the oil products. By utilizing evaluation rules computed on the cloud server, the module calculates the short-term and long-term results based on the data collected by the AIoT module. The short-term results represent the evaluation outcome derived from the current collected data, mainly reflecting the assessment of the current batch of oil products. On the other hand, the long-term results integrate historical data of sesame oil, including the current data, to provide insights into the stability of the sesame oil over time.

The data collected by the ISO Evaluator are securely stored, ensuring the integrity, accuracy, and traceability of information throughout the supply chain. This feature empowers individuals to easily verify the authenticity and quality of sesame oil by accessing the recorded data. Users can conveniently access the system through their computer or mobile devices, enhancing the user-friendly nature of the ISO Evaluator. The system’s functionality enables users to effortlessly view sesame oil-related data collected by the AIoT module and obtain evaluation results. This seamless access allows users to review the collected sesame oil data and access evaluation results, thereby enhancing the overall user experience of the ISO Evaluator.

### 2.2. Cloud Service Platform

The Cloud Service Platform plays a crucial role in managing and processing data collected from multiple ISO Evaluators. It serves as a centralized hub that offers scalability and flexibility to handle a large volume of data, perform in-depth analysis using deep learning techniques, and generate high-quality assessment results. Additionally, the platform enables data visualization, reporting, and remote monitoring, making the system more user-friendly and accessible. At the core of the Cloud Service Platform is deep learning, ensuring the security and integrity of uploaded data. Furthermore, deep learning is employed to analyze the data and establish rules, which are subsequently transmitted back for use by the ISO Evaluator. The platform is divided into two components: (i) the data normalization module and (ii) the deep learning module. The following sections will elaborate on each component.

#### 2.2.1. Data Normalization

The primary responsibility of the data normalization module is to categorize and format data in order to create a computational dataset that can be conveniently utilized by subsequent deep learning modules. This integrated dataset encompasses gas sensor data uploaded from the ISO Evaluator, sesame oil color images, and lipid quality indicators. Furthermore, due to potential variations associated with the sensors, it is essential to apply initial filtering, formatting, and dataset generation to data uploaded by different ISO Evaluators. This process ensures consistent data formatting and ultimately enables the creation of a computational dataset.

After obtaining the sensed values, they undergo min–max scaling normalization [38]. This process involves computing the difference between each original data point and the minimum value in the dataset, followed by dividing this difference by the range between the maximum and minimum values. This scaling procedure, represented by Equation (1), transforms all data points into a standardized range between 0 and 1. This normalization step is employed to enhance the training efficiency of subsequent models. For each data point *x*, you can calculate its scaled value *x_scaled_* by subtracting *x_min_* from *x* and dividing the result by the range between *x_max_* and *x_min_*.

(1) $$x_{scaled}=\frac{x-x_{min}}{x_{max}-x_{min}}$$

where *x* represents an individual data point, *x_min_* represents the minimum value in the original dataset, and *x_max_* represents the maximum value in the original dataset.

#### 2.2.2. Deep Learning Module

**Figure 3:**
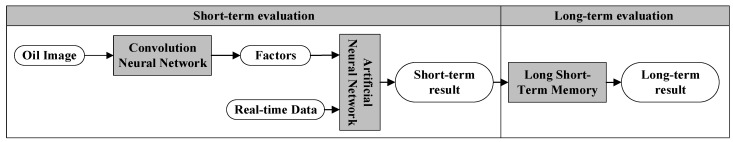
The working flow of the deep learning module.

The deep learning module plays a crucial role in training and establishing evaluation criteria. Furthermore, deep learning has recently made significant advancements in food fraud and food quality. Convolutional Neural Networks (CNN) can be employed to analyze the adulteration of coffee or saffron and to examine the food matrix [39,40,41]. Artificial Neural Networks (ANN) extract relevant factors and efficiently classify data, for instance, in the analysis of food adulteration and quality [42,43,44]. If there are correlations between the data, Long Short-Term Memory (LSTM) networks are utilized, as demonstrated in studies that apply LSTM to assess the quality of oil and meat products [45,46,47]. Within this module, a Convolutional Neural Network (CNN) is utilized to analyze color images of sesame oil. The output from the CNN is then combined with gas sensor data to establish a short-term evaluation criterion using an Artificial Neural Network (ANN). For the long-term evaluation criterion, historical data are analyzed using Long Short-Term Memory (LSTM). In the following sections, it provides operation modes for (a) Convolutional Neural Network (CNN), (b) Artificial Neural Network (ANN), and (c) Long Short-Term Memory (LSTM). The working flow is shown in Figure 3.

(a) Convolutional Neural Network

Convolutional Neural Networks (CNN) play a crucial role in sesame oil detection. CNN utilizes various features to compare different parts of the image and calculates the similarity between each part and its corresponding features. It consists of four main units: convolution layers, pooling layers, rectified linear units (ReLUs) layers, and fully connected layers [48].

The convolution layers apply convolutional kernels or filters to the input image, generating feature maps. The pooling layers, commonly used after the convolution layers, reduce the size of feature maps by selecting either the maximum or average value within each region. The rectified linear units (ReLUs) layers apply the rectified linear unit function to the input elements, effectively solving the problem of gradient vanishing during training. Finally, the fully connected layers connect all neurons from the previous layer to the current layer, enabling global feature extraction and prediction.

**Figure 4:**
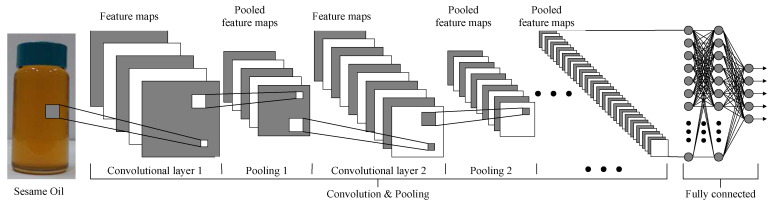
The working flow of the Convolutional Neural Network.

By combining these layers, CNN effectively analyzes different parts of the sesame oil image and extracts relevant information related to its features. This approach of establishing the association between CNN and sesame oil enhances our understanding of sesame oil characteristics and quality, providing accurate and reliable results for sesame oil detection. The detail working flow is shown in Figure 4.

(b) Artificial Neural Network

**Figure 5:**
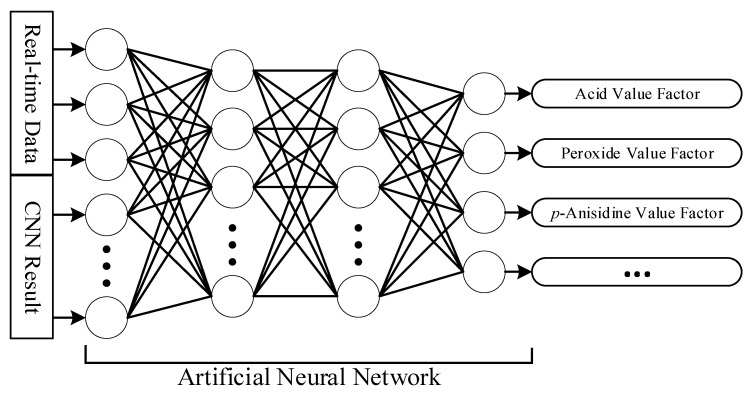
The Artificial Neural Network for indicators.

An Artificial Neural Network (ANN) is constructed by combining multiple artificial neurons. The operation of an artificial neuron involves multiplying the values of all connected nodes from the previous layer by the strengths of their corresponding edges. The resulting values are then summed, and after passing through an activation function, the output of the artificial neuron is obtained. Figure 5 illustrates the ANN architecture used in this study.

The input data to the ANN consists of factors from the CNN and the sensor responses of MQ-2, MQ-3, MQ-4, MQ-5, MQ-6, MQ-7, MQ-8, MQ-9, MQ-135, and MQ-137. The output layer consists of calculated short-term parameters such as acid value, peroxide value, and *p*-anisidine value for sesame oil. Ultimately, the ANN is used to establish a short-term evaluation criterion for frontend applications.

(c) Long Short-Term Memory

The Long Short-Term Memory (LSTM) is used for analyzing time series data, capturing long-term temporal dependencies, and enabling prediction and classification tasks.

**Figure 6:**
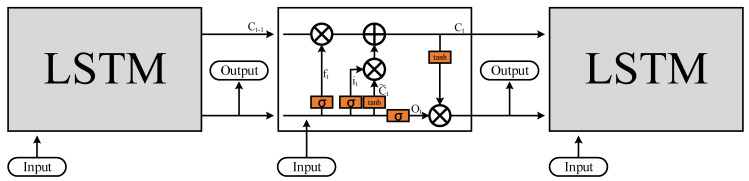
The working flow of the Long Short-Term Memory model.

In this study, LSTM is employed to evaluate the preservation time of sesame oil, considering the correlation between time and data by combining past event data. Therefore, the LSTM is utilized to address the issue of capturing long-term patterns in the data. The LSTM model is shown in Figure 6. The following steps will explain the LSTM model in detail.

The first step is to determine whether information needs to be forgotten. This decision is made by the forget gate, represented by *σ*, as shown in Equation (2). It calculates the sigmoid value based on the previous output and the current input. The resulting value ranges between 0 and 1. If *f_t_* is 1, it means that the memory is fully retained, while a value of 0 indicates complete forgetting.

(2) $$f_{t}=\sigma (W_{f}\cdot [h_{t-1},x_{t}]+b_{f})$$

where *f_t_* represents the forget gate value at time step *t*, *σ* represents the sigmoid activation function, *W_f_* is the weight matrix associated with the forget gate, [*h_t_*_−1_, *x_t_*] represents the concatenation of the previous output *h_t_*_−1_ and the current input *x_t_*, and *b_f_* is the bias term associated with the forget gate.

The second step is to record the input data into the main cell state. This involves two sub-steps. Firstly, the input gate represented by *σ* determines which values should be updated. Similar to the forget gate, the value ranges between 0 and 1. If *i_t_* is 1, it means complete replacement, while a value of 0 means no replacement. The equation is shown in Equation (3). Next, a new candidate value, ${\tilde{C}}_{t}$, is created using the hyperbolic tangent (tanh) function, which will be used to update the subsequent cell state. The equation is shown in Equation (4).

(3) $$i_{t}=\sigma \left(W_{i}\cdot [h_{t-1},x_{t}]+b_{i}\right)$$

(4) $${\tilde{C}}_{t}=\tanh\left(W_{C}\cdot [h_{t-1},x_{t}]+b_{C}\right)$$

The third step involves combining the previous state, *C_t_*_−1_, with the candidate value, ${\tilde{C}}_{t}$, to create a new state, *C_t_*. With the parameters calculated in the previous steps, a simple operation is performed to obtain the new state, *C_t_*. The previous state, *C_t_*_−1_, is multiplied by the value obtained from the forget gate(*f_t_*) to determine the influence of the previous memory. Then, it is added to the product of the candidate state, ${\tilde{C}}_{t}$, and the value obtained from the input gate (*i_t_*) to determine the current cell state. The equation is shown in Equation (5).

(5) $$C_{t}=f_{t}\times C_{t-1}+i_{t}\times {\tilde{C}}_{t}$$

Finally, the content to be output is determined. Firstly, the *σ* function is applied to calculate the value of *o_t_* based on the previous output and the current input, as shown in Equation (6). Then, it is multiplied by tanh (*C_t_*) to determine the value to be output in the current step, as shown in Equation (7).

(6) $$o_{t}=\sigma \left(W_{o}\times [h_{t-1},x_{t}]+b_{o}\right)$$

(7) $$h_{t}=o_{t}\times \tanh\left(C_{t}\right)$$

Through the aforementioned process, the Long Short-Term Memory (LSTM) neural network can capture long-term and important information, understanding the historical short-term values. This enables the establishment of long-term estimation rules for use of the ISO Evaluator.

Ultimately, the cloud server will transmit the short-term estimation rules, which are used to understand the current state, and the long-term estimation rules, which are used to understand the long-term supply status. These rules are generated by the deep learning module and provided to the oil quality standard collection module, allowing users to conveniently infer the status of the desired white sesame oil.

## 3. Results and Implementation

This section focuses on the implementation of the hardware and system for the Sesame Oil Quality Assessment Service Platform. The Intelligent Sesame Oil Evaluator (ISO Evaluator) is used for assessing sesame oil quality. The goal is to explore the relationship between the data collected from gas sensors and the color changes of sesame oil measured using traditional oil quality testing methods. The section is divided into two parts: (1) Intelligent Sesame Oil Evaluator and (2) Cloud Service Platform. Each part will be discussed separately.

### 3.1. Intelligent Sesame Oil Evaluator

This study analyzes the traditional testing methods applied to sesame oil stored for different durations, while simultaneously constructing and utilizing sensors to collect data on volatile gases and changes in oil color. Based on this data, an evaluation model is established. This section is divided into three parts: (i) construction of the AIoT module, (ii) determination of traditional oil quality, and (iii) establishment of the evaluation model.

#### 3.1.1. Construction of the AIoT Module

The AIoT module is divided into three parts: (a) sample preparation, (b) device construction, and (c) data collection.

(a) Sample Preparation

The I-Mei 100% pure sesame oil (I-Mei Foods Co., Ltd., Taipei, Taiwan) is divided and sealed in five 50 mL plastic bottles, covered with aluminum foil to avoid light exposure. The measurement of lipid oxidation stability is typically performed under accelerated conditions (60 °C) since it would take a significantly longer time at room temperature for lipid oxidation to occur [49,50,51]. Therefore, a storage temperature of 60 °C is chosen for the samples. Every week, one bottle of sample is taken out and transferred to a −20 °C freezer for storage. In total, samples are stored at 60 °C for between 1 and 9 weeks.

(b) Device construction

**Figure 7:**
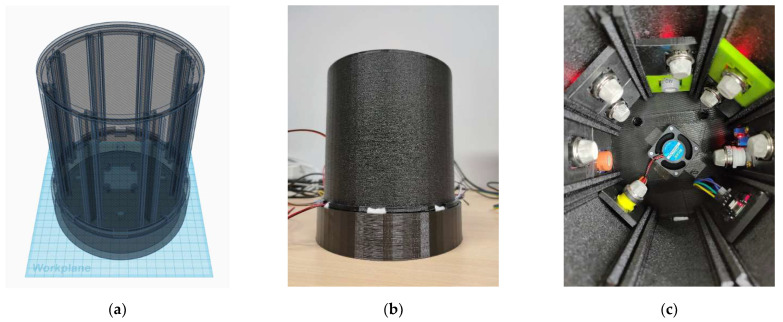
(**a**) The designed sensor house; (**b**) external appearance; (**c**) interior.

The housing of the gas sensor used in this study is 3D-printed using the ATOM 2.0 3D printer (LAYER ONE CO., LTD., Taipei, Taiwan). Figure 7a presents an exploded view of the design, showing the internal components. The sensor housing is 4^2^ × π × 22.5 (cm^3^) which is divided into two layers: the sample layer and the sensor layer. These are separated by a middle partition. The sample layer features eight tracks to securely hold the sensors in place. In the middle of the partition, there is a square opening designed to attach a fan, which accelerates the flow of gas from the sample layer into the sensor layer. Additionally, there are four holes around the opening to allow the airflow from the sensor layer to circulate back to the sample layer, ensuring proper air circulation within the sensor. The sample layer is dedicated to holding the test samples.

Figure 7b depicts the external appearance of the sensor, while Figure 7c illustrates its interior structure. The middle partition is equipped with a 12 V fan, and a total of ten sensors are installed in the sensor layer. These sensors include MQ-2, MQ-3, MQ-4, MQ-5, MQ-6, MQ-7, MQ-8, MQ-9, MQ-135, and MQ-137. They are securely mounted on eight tracks within the sensor layer. An Arduino Mega 2560 control board is used to receive the voltage outputs from these ten different sensor models. As for the collection of sesame oil color images, a Logitech C922 Pro HD Stream Webcam is connected to a Raspberry Pi for capturing the images.

(c) Data Collection

**Figure 8:**
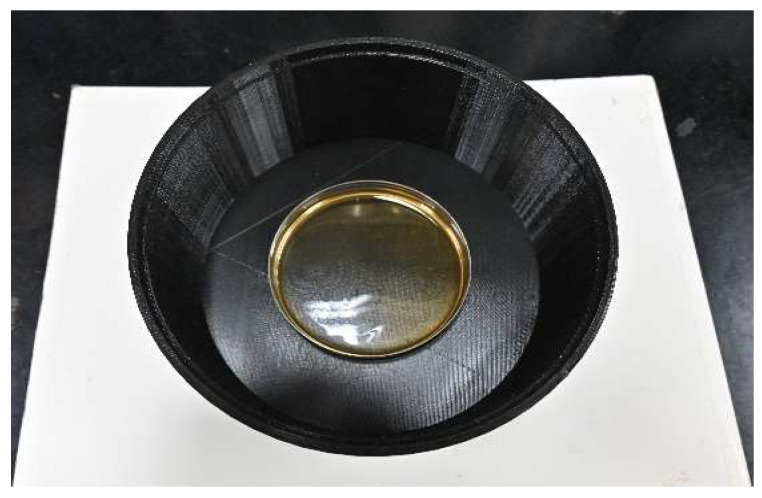
Sesame oil placement test.

The data collection process consists of three stages: a 10-min preheating stage, a 20-min sample equilibration stage, and a 20-min recovery stage. For sample preparation, 3 g of sesame oil is transferred to a plastic culture dish with a diameter of 9 cm and covered with a lid before being placed in the sample layer, which is shown in Figure 8. After the 10 min preheating stage, the sample is inserted into the sensor layer and left for 20-min to allow for gas equilibrium to occur within the sensor. Subsequently, the sample is removed, and a 20-min recovery stage follows to bring the sensor readings back to the baseline, thus completing one measurement.

The sensor sampling frequency is set at one data point per second, resulting in a total of 3000 data points for each measurement. In this study, the average value of the data during the 60-s before the sample equilibration stage is considered as the pre-reaction baseline value. The average value of the data during the 60-s before the recovery stage is considered as the post-reaction equilibrium value. By subtracting these two average values, the individual sensor’s variation in sesame oil odor is obtained as the numerical value for subsequent calculations.

The selection of a 60-s interval for averaging is based on the need to stabilize the sensor output, as there may still be fluctuations after stabilization. Taking the average value over a 60-s interval helps to mitigate the impact of fluctuations on the final calculated results.

**Figure 9:**
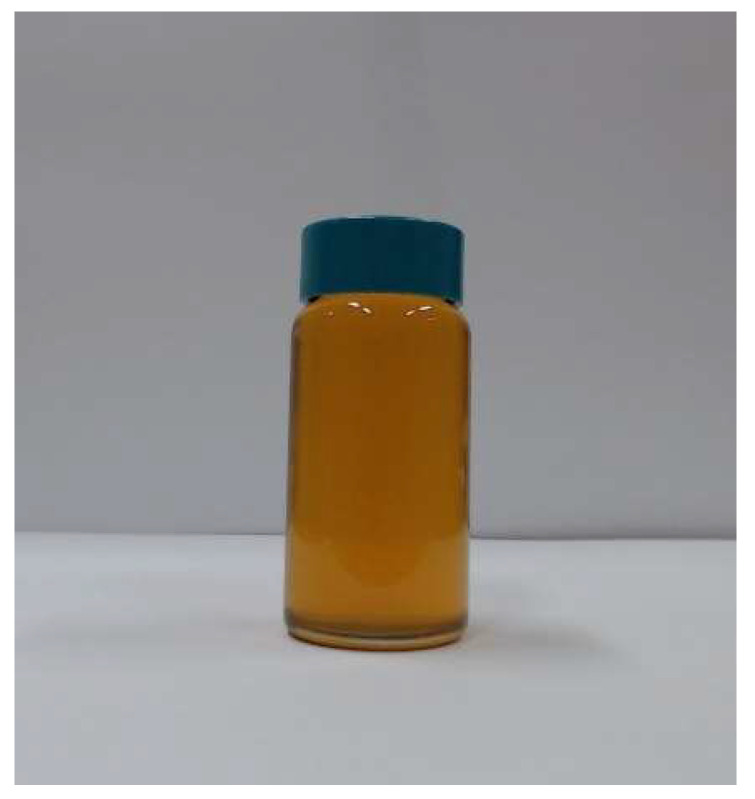
A 25 mL sesame oil sample.

Regarding image capture, the procedure involves filling a sample bottle with 25 mL of sesame oil, as shown in Figure 9. The purpose is to simulate the bottleneck portion of the original bottled oil. The diameter of the bottleneck is chosen to be 3 centimeters, matching the diameter of the sample bottle. This simulation approach is adopted for image recognition purposes.

#### 3.1.2. Determination of Traditional Oil Quality

**Figure 10:**
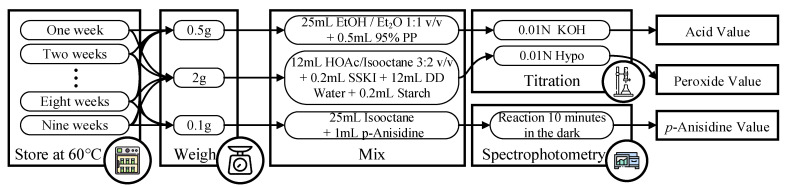
The traditional oil quality determination experiment flow chart.

Three indicators obtained through traditional chemical analysis methods are used: acid value, peroxide value, and *p*-anisidine value. Figure 10 illustrates the experimental procedure integrating these three measurement methods [52]. Detailed explanations of the experimental steps for each of the three indicators will be provided below.

(a) Acid Value

The acid value (AV) is determined by directly titrating the oil/fat in an alcoholic medium against standard potassium hydroxide/sodium hydroxide solution. This method is referenced the Food Additives Specification Test Methods by the Food and Drug Administration, Taiwan [53]. Approximately 0.5 g of oil was weighed and mixed with a mixture of ethanol and ether in a ratio of 1:1 (*v*/*v*) with a volume of 25 mL. Then, 0.5 mL of phenolphthalein indicator was added. The solution was titrated with 0.01 N potassium hydroxide (KOH) ethanol solution until the solution turned pink. A blank test was also performed for calibration. The calculation equation is shown in Equation (8).

(8) $$\mathrm{Acid}\;\mathrm{value}=\frac{N\times V\times 56.11}{W}$$

where *N* is the concentration of the KOH ethanol solution; *V* is the volume of the KOH ethanol solution used for titration (mL); *W* is the weight of the oil (g).

(b) Peroxide Value

The peroxide value (POV) is determined by measuring the amount of iodine which is formed by the reaction of peroxides (formed in fat or oil) with iodide ion. This method is referencd by the Association of Official Agricultural Chemists (A.O.A.C.) [54]. A measure of 2 g of oil was taken and mixed with a mixture of glacial acetic acid and isooctane in a ratio of 3:2 (*v*/*v*) with a volume of 12 mL. After shaking for 30 s, 0.2 mL of saturated potassium iodide solution was added. The mixture was shaken for 1 min, followed by the addition of 12 mL of ultrapure water and 0.2 mL of 1% starch solution. After shaking for 30 s, the solution was titrated with 0.01 N sodium thiosulfate until clarification. The calculation equation is shown in Equation (9).

(9) $$\mathrm{Peroxide}\;\mathrm{value}=\frac{S\times N\times 1000}{W}$$

where *S* is the volume of sodium thiosulfate used for titration (mL); *N* is the concentration of sodium thiosulfate equivalent; *W* is the weight of the oil (g).

(c) *p*-anisidine Value

The *p*-anisidine value (AnV) serves as an indicator of oil oxidation by measuring the total amount of aldehydes present in the oil sample. This method is referenced by the Association of Official Agricultural Chemists (A.O.A.C.) [54]. Approximately 0.1 g of oil was weighed and mixed with isooctane in a volume of 25 mL. Then, 5 mL of this oil solution was taken and mixed with 1 mL of *p*-anisidine reagent (0.25 g of *p*-anisidine dissolved in 100 mL of glacial acetic acid). The mixture was stirred well and left to stand for 10 min. The absorbance value was measured at 350 nm. The calculation equation is shown in Equation (10).

(10) $$p\mathrm{-anisidine}\;\mathrm{value}=\frac{V\times (1.2As-Ab)}{W}$$

where *V* is the volume of isooctane added (mL); *As* is the absorbance value of the sample with *p*-anisidine reagent added; *Ab* is the absorbance value of the sample without *p*- anisidine reagent added; *W* is weight of the oil (g).

(d) Arrhenius Equation

The Arrhenius equation is for the temperature dependence of reaction rates, which is employed in sesame oil preservation experiments to understand how temperature affects its shelf life. This study uses it accelerated aging process of sesame oil. Table 1 illustrates the edible oil quality indexes of sesame oil, including POV, AV, and AnV. The weeks of this table denote that the actual storage time heated to 60 °C and the estimation of shelf life by Arrhenius equation, which is shown as Equation (11) [55].

(11) $$k=Ae^{\frac{-Ea}{RT}}$$

where *k* is the rate constant; *T* is the absolute temperature; *A* is the pre-exponential factor; *Ea* is the activation energy for the reaction; *R* is the universal gas constant.

**Figure 11:**
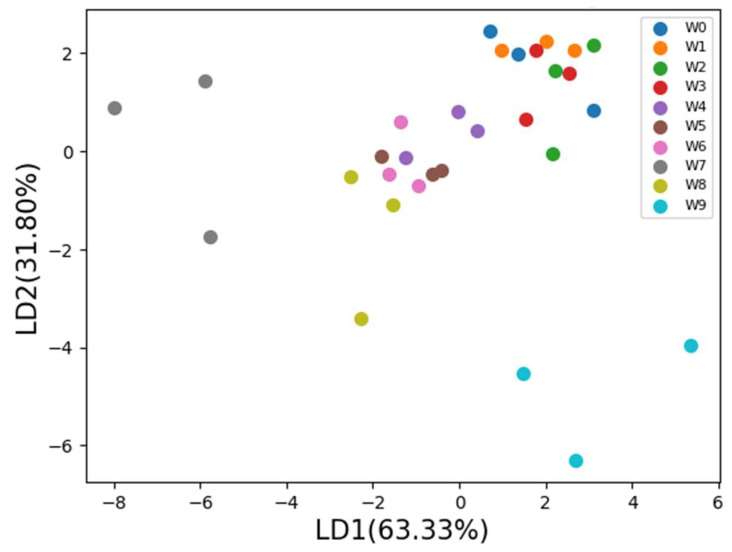
Sensed data are analyzed using LDA.

After heating at 60 °C, the experimental correspondence is illustrated in Table 1 and Table 2. Table 1 indicates that the peroxide value (POV) index of sesame oil after the ninth week has exceeded the standard by 15 meq/kg [35,36]. It represents that the quality of the sesame oil sample began to decline. The acid value and *p*-anisidine value increased slightly but the magnitude was not significant. Table 2 shows that data are sensed by different gas sensors. The sesame oil quality analysis is conducted through linear discriminant analysis (LDA) using sensed data from ten gas sensors. Figure 11 illustrates that the LDA can distinguish sesame oil of different periods by 95.13%, which for LD1 is 63.33% and for LD2 is 31.80%.

To assess the current quality and long-term stability of sesame oil, deep learning models including convolutional neural networks, artificial neural networks, and long short-term memory models are utilized.

Firstly, the convolutional neural network processes the images of sesame oil captured by a webcam. The obtained results are then combined with the response values from 10 different gas sensors and fed into the artificial neural network model. The model outputs three quality indicators: peroxide value, acid value, and *p*-anisidine value. These are used to evaluate the current quality of sesame oil. The evaluation criteria are based on the standards for edible oils [35,36]. If the peroxide value exceeds 15 meq/kg or the acid value exceeds 4 KOH mg/g, then the oil is deemed unsatisfactory. The Long Short-Term Memory model uses the input data from the current sensors to calculate the future changes in the quality of sesame oil, thereby determining its stability. If the predicted values are close to the standards, then the user is notified that they should use the oil as soon as possible.

### 3.2. Cloud Service Platform

The cloud service platform is based on data storage and utilizes a data normalization module to exclude abnormal values and generate a dataset suitable for model training. This section introduces three components: (i) the data normalization module and (ii) the deep learning module.

#### 3.2.1. Data Normalization Module

In a single measurement, each sensor generates 3000 data points, with a total of 10 sensors, resulting in 30,000 data points. Using the Pandas module in Python, the data from the 540–600th data points and the 1200–1740th data points of each sensor are stored in Dataframe format. Then, the Dataframe is converted to Numpy array format using the Numpy module. The mean function from the statistics module is applied to calculate the average value. Finally, the voltage difference between the pre- and post-reaction of each sensor is calculated.

To scale the data generated by each sensor, the MinMaxScaler function from the Sklearn module’s preprocessing stage is used. This function performs min–max scaling, which scales the data between 0 and 1, ensuring that the data are uniformly scaled across all sensors.

#### 3.2.2. Deep Learning Module

In the deep learning module, the Convolutional Neural Network (CNN) is responsible for classifying the color of sesame oil. It analyzes the visual characteristics of the oil sample and categorizes it based on its color properties. The Artificial Neural Network (ANN) is responsible for analyzing the data from the sensors. It processes the sensor data and estimates the current values of peroxide value, acid value, and *p*-anisidine value of the sesame oil. The Long Short-Term Memory (LSTM) module is responsible for analyzing the sensor data and predicting potential future changes in quality. It is particularly effective in capturing temporal dependencies and patterns in the data, enabling it to forecast variations in the quality of the oil.

(a) Convolutional Neural Network

The Convolutional Neural Network (CNN) used in this case is based on the You Only Look Once (YOLO) version seven and implemented in Python 3.8.16. The dataset consists of 50 images, which are divided into 5 classes representing storage at 60 °C from the 1st to 9th week. The training set contains 40 images, and the test set contains 10 images, with a ratio of 8:2. The batch size is set to 5, and the number of epochs is set to 100.

Batch size refers to the grouping or batching of data for training in a neural network. In this case, with a batch size of five, only five data points are inputted into the neural network at a time. After this process is repeated 10 times, it completes one epoch. An epoch is defined as one complete pass through the entire training dataset. In this scenario, when all 50 images have been trained on the neural network, it completes one epoch. The training process is repeated 100 times, corresponding to 100 epochs, to complete the training.

LabelImg is a tool used for annotating the position and names of objects in images. When implementing object detection using deep learning, a large dataset with known labels is required. This dataset consists of images with objects annotated with their respective positions and names. LabelImg is a manual tool commonly used for this annotation process.

When evaluating the accuracy of the trained model, precision and recall are used as evaluation metrics. Precision calculates the proportion of true positive predictions out of all positive predictions, where TP represents true positive (the predicted result is the class of interest and matches the ground truth) and *FP* represents false positive (the predicted result is the class of interest but does not match the ground truth). Recall calculates the proportion of true positive predictions out of all actual positive instances, where *FN* represents false negative (the predicted result is not the class of interest but should be) and *TN* represents true negative (the predicted result is not the class of interest and matches the ground truth). Precision is calculated using Equation (12), while recall is calculated using Equation (13).

(12) $$Precision=\frac{TP}{TP+FP}$$

(13) $$Recall=\frac{TP}{TP+FN}$$

This study sets the batch size to 11 and the learning iterations to 100 epochs. Learning iterations exceeding 100 epochs would lead to overfitting in the image recognition model, thereby causing a decrease in recognition performance.

The performance in classifying the results for each week shows an average precision of 64.4% and an average recall of 55.6%. The results show that the image recognition model has shown initial performance. However, due to the limitations in dataset size and source, there exists an opportunity for optimization in future research.

(b) Artificial Neural Network

In the ANN model used in this study, the Mean Absolute Percentage Error (MAPE) is employed as the loss function. Its calculation is defined by Equation (14).

(14) $$MAPE=\frac{100\%}{n}\sum_{t=1}^{n}\left|\frac{A_{t}-F_{t}}{A_{t}}\right|$$

where *A_t_* represents the actual value and *F_t_* represents the predicted value. In the equation, the index *t* starts from 1 and goes up to *n*, indicating that MAPE calculates the sum of percentage errors from the first predicted value to the *n*th predicted value. This sum is then divided by the total number of predicted values *n* to obtain the average.

During the training process, a total of 40 data points were used (8 data points per week). The training set and test set were split in an 8:2 ratio. The batch size was set to 8. After training the model for 1000 iterations, the MAPE for the prediction results was found to be 8.1820%.

(c) Long Short-Term Memory

The Long Short-Term Memory (LSTM) model is developed to predict sesame oil storage weeks. The environment for this model is Python, primarily leveraging the Keras library for LSTM model development.

In the first phase, LSTM serves as the neuron, with the input_shape parameter as the LSTM input. There are two arguments, which are n_step and n_feature. "n_step" specifies the number of preceding data points used for prediction, set to 6, signifying that the previous data group is utilized for prediction. "n_feature" denotes the feature size, set to 10, because there are 10 sensors. Since these sensors measure various values, each prediction point takes input from these 10 sensor readings for prediction. In the final phase, traditional neurons are employed to calculate values from the preceding LSTM phase and produce the result.

**Figure 12:**
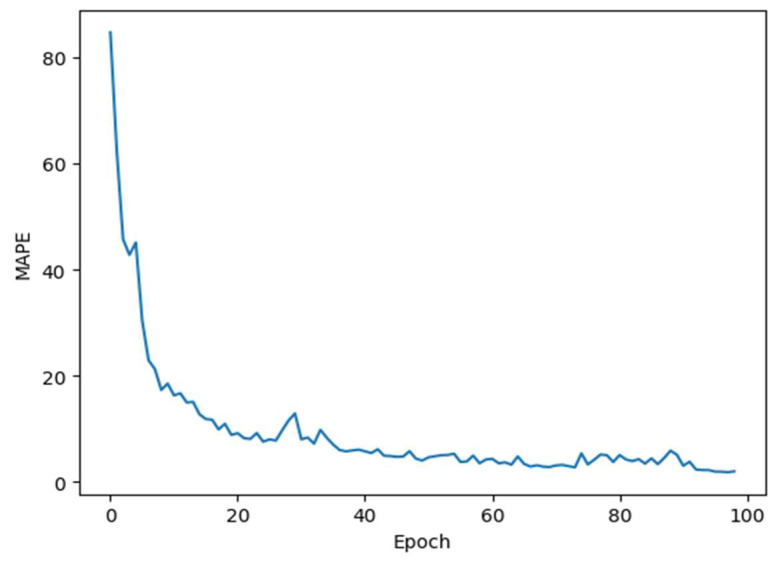
The Mean Absolute Percentage Error value for each epoch.

In the training phase, it sets the batch size of 10, and the epoch of 100 iterations, and evaluated the model performance by the Mean Absolute Percentage Error (MAPE). In this study, the MAPE value for the training result of the LSTM model was 1.99%. The training MAPE value is shown in Figure 12. The MAPE value is 0.44%, indicating that the results computed by the LSTM model closely align with the actual outcomes.

**Figure 13:**
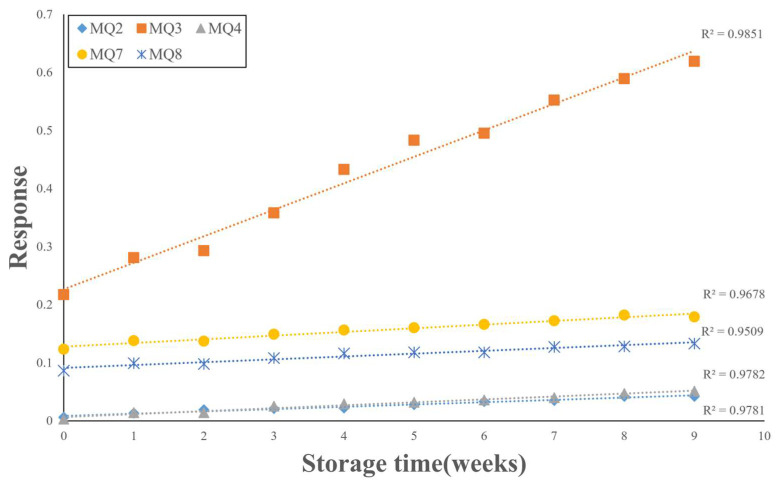
Calibration curves of MQ2, MQ3, MQ4, MQ7, and MQ8.

After sensed data are normalized to a value between 0–1 through the min–max scaling method, results indicate that MQ2, MQ3, MQ4, MQ7, and MQ8 sensors are with positive correlation. The min–max scaling method performs a linear transformation on the original data by Equation (3) [38]. Figure 13 shows the calibration curves of MQ2, MQ3, MQ4, MQ7, and MQ8. Hence, the calibration curve with R^2^ is greater than 0.95. According to sensed data and chemical experiment data in the edible oil quality collection module, two rules are reasoned, as follows.

(1) When the value of MQ2 is greater than 0.035, the value of MQ3 is greater than 0.552, the value of MQ4 is greater than 0.04, the value of MQ7 is greater than 0.172, and the value of MQ8 is greater than 0.127, the oil quality has begun to decline.
(2) When the value of MQ2 is greater than 0.042, the value of MQ3 is greater than 0.619, the value of MQ4 is greater than 0.051, the value of MQ4 is greater than 0.179, and the value of MQ8 is greater than 0.133, the POV indicator of oil products may have begun to exceed the standard.

**Figure 14:**
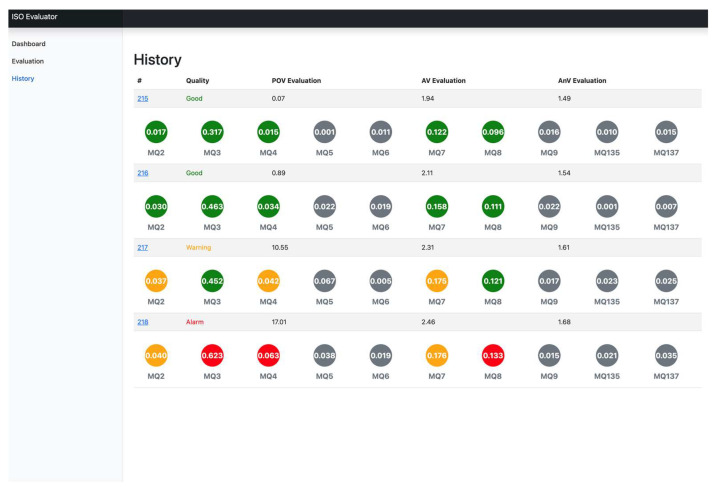
The system interface of the history.

**Figure 15:**
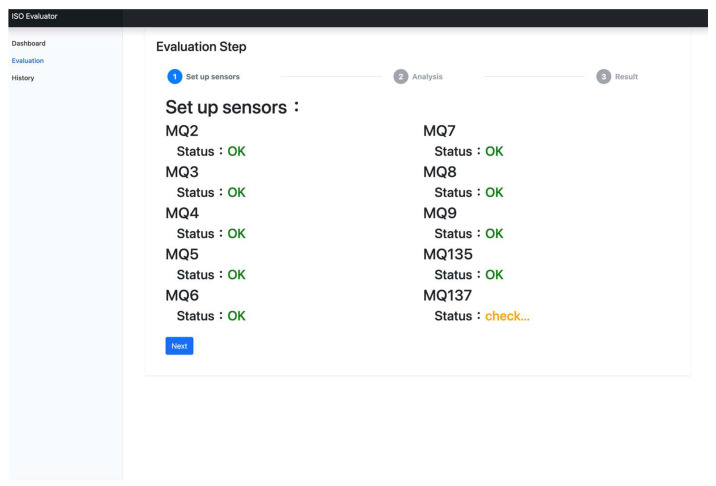
The system interface of gas sensors.

**Figure 16:**
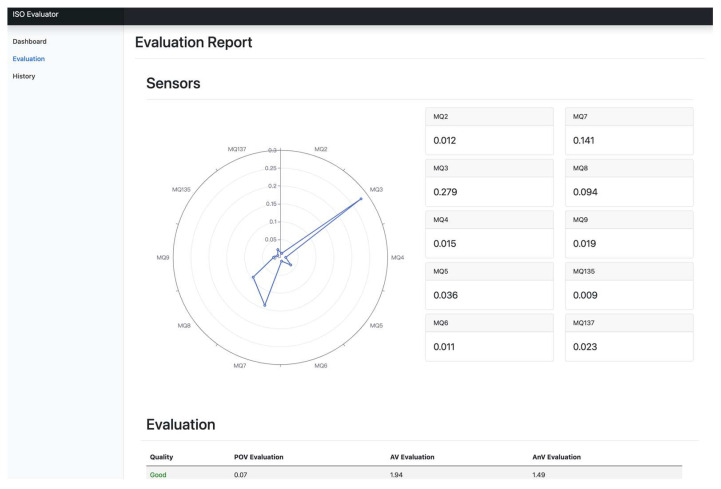
The system interface of an evaluation report.

After reasoning out evolution rules, the proposed Sesame Oil Quality Assessment Service Platform is designed and implemented. Figure 14, Figure 15 and Figure 16 are the designed system interfaces. Figure 14 is the history of each detection. Figure 15 is the function of sesame oil detection. Figure 16 shows an evaluation report.

## 4. Conclusions

This study designs and proposes a Sesame Oil Quality Assessment Service Platform, which is composed of an Intelligent Sesame Oil Evaluator (ISO Evaluator) and a Cloud Service Platform. Users can quickly assess the quality of the sesame oil using this platform. The ISO Evaluator employs Artificial Intelligence of Things (AIoT) sensors to detect changes in volatile gases and the color of the oil during storage. The data calculated in this study led to the following conclusions:

(1) The sensor data from MQ2, MQ3, MQ4, MQ7, and MQ8 sensors were used to analyze the oil samples at different weeks. The data exhibited a strong linear relationship with an R-squared value greater than 0.95. This indicates that these five sensors can effectively detect variations in oil quality across different weeks. To tackle the quality assessment challenge, a combination of traditional indicators such as acid value, peroxide value, and *p*-anisidine, along with AIoT gas sensors and webcams, can be utilized. The recorded data can then be analyzed using ANN, CNN, and LSTM models for comprehensive analysis of oil quality.
(2) Regarding the analysis of oil samples at different weeks, it was observed that when analyzing oil images solely through CNN, due to data quantity and design limitations, the performance resulted in an average precision of 64.4% and an average recall of 55.6%. These results suggest that there is room for improvement when using image analysis alone.
(3) By combining the features analyzed using CNN with sensor data and using ANN for analysis, the performance yielded a Mean Absolute Percentage Error (MAPE) of 8.1820%. This demonstrates that the integration of CNN and sensors can effectively enhance the recognition accuracy.
(4) When analyzing the oil samples recorded over an extended period along with sensor data using LSTM, the performance achieved a MAPE of 0.44%. This implies that the results produced by LSTM closely match the actual conditions, indicating that LSTM can effectively distinguish the quality status of oil samples at different weeks by utilizing oil images and sensor data recorded over a long-term period.

Four problems are considered and overcome: digitization, quality assessment, quality monitoring, and scalability.

(1) To tackle the digitization problem, the use of AIoT technologies can be implemented for efficient and secure data recording and management.
(2) To tackle the quality assessment challenge, a combination of traditional indicators such as acid value, peroxide value, and *p*-anisidine, along with AIoT gas sensors and webcams, can be utilized. The recorded data can then be analyzed using ANN, CNN, and LSTM models for comprehensive analysis of oil quality.
(3) To overcome the quality monitoring issue, the application of LSTM can enable continuous monitoring and analysis of sesame oil quality throughout the supply chain.
(4) To enhance Scalability, the development platform can be designed as an open system, allowing for the seamless integration of additional AIoT sensors and the incorporation of new services to meet evolving needs.

The integration of AIoT, CNN, and LSTM technologies in the Sesame Oil Quality Assessment Service Platform holds great potential for enhancing consumer trust, ensuring food safety, and maintaining product integrity. Future research directions can focus on further advancements in deep learning, multi-modal sensing, real-time monitoring, data-driven decision support, and blockchain applications to continually improve the platform’s capabilities and address emerging challenges in the oil industry.

## Figures and Tables

**Table 1:** Edible oil quality indexes.

	Quality	POV ^b^	AV ^b^	AnV ^b^
Weeks				
0	(0.0) ^a^	0.01 ± 1.49	1.95 ± 0.11	1.58 ± 0.02
1	(5.5) ^a^	0.08 ± 0.28	1.93 ± 0.12	1.56 ± 0.01
2	(10.9) ^a^	0.45 ± 0.26	1.97 ± 0.10	1.60 ± 0.03
3	(16.4) ^a^	0.82 ± 0.28	2.02 ± 0.08	1.64 ± 0.06
4	(21.8) ^a^	2.45 ± 0.23	2.14 ± 0.05	1.64 ± 0.05
5	(27.4) ^a^	4.09 ± 0.17	2.26 ± 0.02	1.63 ± 0.05
6	(32.8) ^a^	7.42 ± 0.15	2.28 ± 0.04	1.62 ± 0.06
7	(38.3) ^a^	10.75 ± 0.13	2.30 ± 0.07	1.60 ± 0.06
8	(43.7) ^a^	13.85 ± 0.16	2.37 ± 0.09	1.63 ± 0.05
9	(49.2) ^a^	16.95 ± 0.19	2.44 ± 0.10	1.67 ± 0.03

^a^ Predictions of parameters at 25 °C based on the Arrhenius equation. ^b^ Mean ± SD (*n* = 3).

**Table 2:** The data sensed using gas sensors.

	Sensors	MQ2 ^b^	MQ3 ^b^	MQ4 ^b^	MQ5	MQ6	MQ7 ^b^	MQ8 ^b^	MQ9	MQ135	MQ137
Weeks											
0	(0.0) ^a^	0.006	0.217	0.003	0.001	0.003	0.123	0.086	0.005	0.005	0.005
1	(5.5) ^a^	0.013	0.281	0.014	0.013	0.012	0.138	0.099	0.016	0.008	0.011
2	(10.9) ^a^	0.019	0.293	0.014	0.043	0.016	0.137	0.098	0.017	0.010	0.011
3	(16.4) ^a^	0.021	0.358	0.025	0.063	0.018	0.149	0.108	0.022	0.012	0.009
4	(21.8) ^a^	0.022	0.433	0.029	0.049	0.014	0.156	0.116	0.021	0.006	0.009
5	(27.4) ^a^	0.028	0.483	0.032	0.044	0.009	0.160	0.118	0.018	0.001	0.009
6	(32.8) ^a^	0.033	0.495	0.036	0.058	0.006	0.166	0.118	0.019	0.010	0.018
7	(38.3) ^a^	0.035	0.552	0.040	0.069	0.001	0.172	0.127	0.018	0.019	0.026
8	(43.7) ^a^	0.042	0.589	0.047	0.049	0.006	0.182	0.128	0.015	0.020	0.031
9	(49.2) ^a^	0.042	0.619	0.051	0.020	0.009	0.179	0.133	0.015	0.021	0.035

^a^ Predictions of parameters at 25 °C based on the Arrhenius equation. ^b^ R^2^ is greater than 0.95.

## Data Availability

Data is contained within the article.
